# Supplementary material for: Proinflammatory Dietary Intake is Associated with Increased Risk of Metabolic Syndrome and Its Components: Results from the Population-Based Prospective Study
Source: Nutrients. 2020 Apr 24;12(4):1196. doi: 10.3390/nu12041196 (PMC7230546; doi:10.3390/nu12041196)
Supplement: Supplementary file 1 [file nutrients-12-01196-s001.pdf]

Table S1. Distribution of food parameters and nutrients of Dietary Inflammatory Index (DII®) among cases and controls.

	Normal (n=154,305)	MS (n=3,507)	WC (n=5,885)	TG (n=8,964)	HDL-C (n=3,937)	Glu (n=11,337)	BP (n=10,148)	*p*-value[a]
**Pro-inflammatory**								
Energy (kCal)	1745.8(532.4)	1768.2(537.6)	1753.1(519.3)	1734.2(528.3)	1737.6(533)	1759(528.9)	1744.3(512.1)	0.01
Total fat (g)	138.9(54.1)	135.1(53)	137.2(53.8)	136.6(53.5)	135.9(52.7)	137.3(53)	136.9(52.1)	0.0001
Saturated fat (g)	5.19(2.56)	5.11(2.54)	5.18(2.57)	5.16(2.51)	5.11(2.51)	5.16(2.51)	5.15(2.49)	0.03
Trans fat (g)	0.21(0.17)	0.21(0.18)	0.21(0.18)	0.2(0.18)	0.2(0.17)	0.2(0.17)	0.2(0.17)	0.1
Cholesterol (mg)	92.5(54.2)	89.2(50.9)	92.5(52.5)	92.2(53.7)	90.1(51.6)	92.6(53.1)	92.2(53.2)	0.001
Carbohydrate (g)	716.6(69.9)	720.6(68.8)	718.4(69.6)	718.9(69.8)	720.6(68.1)	718.5(68.6)	718.8(67.3)	0.001
Protein (g)	134.5(26)	133.6(25.6)	134.4(25.5)	134.6(26.4)	133.4(25.1)	134.1(25.7)	134.1(25.2)	0.1
Iron (mg)	5.7(1.8)	5.6(1.8)	5.7(1.8)	5.7(1.8)	5.7(1.8)	5.7(1.7)	5.7(1.7)	0.0003
Vitamin B12 (mg)	3.3(2.1)	3.3(2.1)	3.3(2)	3.4(2.1)	3.3(2)	3.3(2.1)	3.3(2)	0.9
**Anti-inflammatory**								
Flavan-3-ol (mg)	2712.9(5257.6)	2712.2(5345.4)	2644.3(5081.1)	2731.4(5234.7)	2712.4(5224.5)	2629.3(5096.7)	2545.9(4930.1)	0.7
Flavones (mg)	19(21.5)	17.7(18.6)	18.7(19)	20(22.3)	19.4(20.8)	19(20.8)	18.9(20.4)	0.02
Flavonols (mg)	419.4(739.7)	416.7(751.2)	409.9(715.2)	423.7(736.8)	420(735.4)	408(716.4)	396.2(693.9)	0.2
Flavonones (mg)	158.6(222.9)	145(192.8)	153.5(193.3)	168.7(232.9)	161.5(212.3)	158.8(216.2)	156.8(211.9)	0.02
Tea (g)	30.5(59.4)	30.5(60.4)	29.8(57.4)	30.7(59.1)	30.5(59)	29.6(57.5)	28.6(55.7)	0.9
Iso-flavones (mg)	42.9(51.7)	41.5(50.3)	42.1(50.1)	43.3(51.5)	42.9(51)	41.7(49.5)	42.4(49.7)	0.1
MUFA (g)	4.5(2.7)	4.4(2.7)	4.5(2.7)	4.5(2.7)	4.4(2.7)	4.5(2.7)	4.5(2.6)	0.1
PUFA (g)	2.5(1.6)	2.5(1.6)	2.6(1.6)	2.6(1.6)	2.5(1.6)	2.5(1.6)	2.5(1.5)	0.1
Zinc (mg)	4.5(1.1)	4.5(1.1)	4.5(1)	4.6(1.1)	4.5(0.9)	4.5(1)	4.5(1)	0.5
Caffeine (μg)	0.1(0.1)	0.1(0.1)	0.1(0.1)	0.1(0.1)	0.1(0.1)	0.1(0.1)	0.1(0.1)	0.005
Carotene (μg)	1373.2(928.6)	1363.2(930.7)	1371.5(888.5)	1381.4(909)	1369.9(961.8)	1382.2(908.9)	1356.8(892)	0.5
Fiber (g)	3.3(1.3)	3.3(1.3)	3.3(1.3)	3.4(1.3)	3.3(1.3)	3.3(1.2)	3.3(1.2)	0.06
Folic acid (mg)	124.8(53)	122.9(52.2)	124.9(52.4)	125.9(52.8)	124.8(54.1)	124.7(51.6)	123.9(51.5)	0.01
Garlic (g)	0.2(0.5)	0.2(0.4)	0.2(0.5)	0.2(0.5)	0.2(0.5)	0.2(0.5)	0.2(0.5)	0.6
Magnesium (mg)	33.6(16.2)	32.8(15.5)	34.1(15.9)	33.7(15.8)	33.1(15.6)	33.5(15.6)	33.4(15.6)	0.006
N-3 PUFA (g)	0.2(0.2)	0.2(0.2)	0.2(0.2)	0.2(0.2)	0.2(0.2)	0.2(0.2)	0.2(0.2)	0.9
N-6 PUFA (g)	1.4(0.8)	1.3(0.8)	1.4(0.8)	1.3(0.8)	1.3(0.8)	1.4(0.8)	1.3(0.7)	0.07
Niacin (mg)	8.3(1.8)	8.2(1.8)	8.3(1.8)	8.3(1.8)	8.2(1.7)	8.3(1.8)	8.2(1.7)	0.6
Onion (g)	3.7(3.5)	3.9(3.6)	3.9(3.6)	3.8(3.5)	3.8(3.6)	3.9(3.5)	3.8(3.4)	<0.0001
Riboflavin (mg)	0.5(0.2)	0.5(0.2)	0.5(0.2)	0.5(0.2)	0.5(0.2)	0.5(0.2)	0.5(0.2)	0.003
Anthocyanidins (mg)	14.8(13)	14.4(12.7)	15.1(12.7)	15.3(12.8)	15.5(13.7)	14.9(12.5)	15(12.5)	0.1
Selenium (mg)	78.6(10.4)	79.2(10)	78.6(10)	78.6(10.3)	78.4(10.2)	78.8(10)	78.8(10)	0.0001
Vitamin B1 (mg)	0.6(0.1)	0.6(0.1)	0.6(0.1)	0.6(0.1)	0.6(0.1)	0.6(0.1)	0.6(0.1)	0.01
Vitamin A (mg)	273.9(163.3)	269.8(161.9)	272.9(156.2)	274.7(159.2)	272(168)	274.7(159.6)	270.6(156.8)	0.1
Vitamin B6 (mg)	0.9(0.2)	0.9(0.2)	0.9(0.2)	0.9(0.2)	0.9(0.2)	0.9(0.2)	0.9(0.2)	0.1
Vitamin C (mg)	61.5(33.1)	60.1(32.2)	61.9(32.5)	62.8(33.3)	61.7(33.4)	61.4(32.1)	61.2(32.3)	0.01
Vitamin D (mg)	1.8(1.3)	1.7(1.3)	1.8(1.4)	1.8(1.4)	1.7(1.3)	1.7(1.3)	1.8(1.3)	0.06

Vitamin E (mg)	4.6(1.6)	4.5(1.6)	4.6(1.6)	4.6(1.6)	4.6(1.6)	4.6(1.5)	4.6(1.5)	0.0005

Values are presented as mean (standard deviation,[a] Kruskal-Wallis test was used to calculate *p* values between normal and metabolic syndrome. MUFA: monosaturated fatty acid, PUFA, polyunsaturated fatty acid, MS: metabolic syndrome, WC: waist circumference, TG: triglycerides, HDL-C: high density lipid cholesterol, Glu, glucose, BP, blood pressure.

Table S2. Hazard ratios (HRs) and 95% confidence intervals (CIs) for risk of metabolic syndrome for selected food parameters according to the DII® quintiles for all participants

	Quintiles of Dietary Inflammatory Index® (DII®)[a,b,c]					
**Food parameters**	Q1	Q2	Q3	Q4	Q5	*P* for trend[d]
**Pro-inflammatory**						
Energy (Kcal)	Ref.	0.99(0.89-1.11)	1.01(0.91-1.13)	1.06(0.95-1.18)	0.95(0.85-1.06)	0.74
Total fat (g)	Ref.	0.97(0.88-1.08)	1.10(0.99-1.21)	0.93(0.83-1.04)	0.99(0.88-1.10)	0.58
Saturated fat (g)	Ref.	0.99(0.89-1.10)	1.03(0.92-1.14)	0.93(0.83-1.03)	0.96(0.86-1.07)	0.29
Trans fat (g)	Ref.	1.00(0.90-1.12)	0.97(0.87-1.08)	0.99(0.90-1.11)	1.03(0.98-1.15)	0.64
Cholesterol (mg)	Ref.	0.89(0.80-0.99)	0.93(0.84-1.04)	0.99(0.89-1.10)	0.95(0.85-1.05)	0.92
Carbohydrate (g)	Ref.	1.02(0.92-1.14)	1.11(0.99-1.23)	1.02(0.92-1.14)	1.07(0.96-1.20)	0.24
Protein (g)	Ref.	0.94(0.84-1.04)	0.98(0.88-1.09)	0.94(0.85-1.05)	**0.88(0.79-0.98)**	0.04
Iron (mg)	Ref.	1.06(0.96-1.18)	1.05(0.95-1.17)	0.99(0.89-1.10)	**0.80(0.71-0.89)**	0.0001
Vitamin B12 (mg)	Ref.	1.09(0.98-1.21)	1.07(0.97-1.20)	1.01(0.91-1.13)	1.00(0.90-1.11)	0.61
**Anti-inflammatory**						
Flavan-3-ol (mg)	Ref.	1.10(0.98-1.22)	1.09(0.98-1.21)	0.91(0.82-1.01)	**0.83(0.74-0.92)**	<0.0001
Flavones (mg)	Ref.	1.10(0.99-1.22)	1.13(1.01-1.25)	1.11(0.99-1.24)	0.99(0.88-1.10)	0.98
Flavonols (mg)	Ref.	1.02(0.91-1.13)	1.06(0.95-1.17)	**0.89(0.80-0.99)**	**0.79(0.71-0.88)**	<0.0001
Flavonones (mg)	Ref.	1.09(0.98-1.21)	1.18(1.06-1.31)	1.11(0.99-1.23)	1.04(0.94-1.17)	0.35
Tea (g)	Ref.	0.78(0.49-1.25)	1.03(0.94-1.13)	**0.87(0.79-0.95)**	**0.78(0.71-0.86)**	<0.0001
Iso-flavones (mg)	Ref.	1.19(1.07-1.32)	1.08(0.97-1.20)	0.99(0.89-1.10)	**0.87(0.78-0.97)**	0.0002
MUFA (g)	Ref.	1.00(0.90-1.11)	0.98(0.88-1.09)	1.03(0.92-1.14)	0.99(0.89-1.10)	0.86
PUFA (g)	Ref.	0.95(0.85-1.06)	0.97(0.88-1.08)	0.93(0.84-1.04)	**0.82(0.73-0.91)**	0.0008
Zinc (mg)	Ref.	1.04(0.94-1.16)	1.04(0.94-1.16)	0.99(0.89-1.11)	0.99(0.90-1.11)	0.63
Caffeine (µg)	Ref.	0.99(0.89-1.11)	1.12(1.01-1.25)	1.09(0.97-1.21)	1.18(1.06-1.32)	0.0005
Carotene (µg)	Ref.	1.04(0.93-1.16)	1.07(0.96-1.19)	1.00(0.90-1.11)	0.92(0.83-1.03)	0.12
Fiber (g)	Ref.	0.95(0.85-1.05)	0.97(0.87-1.08)	0.93(0.84-1.03)	**0.81(0.72-0.90)**	0.0004
Folic acid (mg)	Ref.	0.98(0.88-1.08)	0.95(0.85-1.05)	0.92(0.82-1.02)	**0.85(0.76-0.95)**	0.002
Garlic (g)	Ref.	0.94(0.84-1.06)	1.05(0.94-1.16)	0.98(0.88-1.09)	0.97(0.88-1.08)	0.84
Magnesium (mg)	Ref.	1.06(0.96-1.18)	1.08(0.97-1.20)	1.12(1.01-1.24)	0.96(0.86-1.07)	0.82
N-3 PUFA (g)	Ref.	1.00(0.90-1.11)	0.98(0.88-1.09)	1.04(0.94-1.15)	**0.86(0.78-0.96)**	0.04
N-6 PUFA (g)	Ref.	1.09(0.98-1.21)	1.13(1.01-1.25)	1.03(0.92-1.15)	1.08(0.97-1.21)	0.39

Niacin (mg)	Ref.	1.15(1.03-1.28)	1.11(0.99-1.23)	1.10(0.98-1.22)	0.99(0.89-1.11)	0.62
Onion (g)	Ref.	1.02(0.91-1.14)	1.14(1.02-1.27)	1.25(1.12-1.39)	1.14(1.02-1.27)	0.0001
Riboflavin (mg)	Ref.	0.96(0.86-1.06)	0.96(0.87-1.07)	0.94(0.84-1.04)	**0.81(0.73-0.91)**	0.0008
Anthocyanidins (mg)	Ref.	1.10(0.99-1.23)	1.09(0.98-1.21)	1.12(1.01-1.25)	1.09(0.98-1.22)	0.11
Selenium (mg)	Ref.	1.02(0.91-1.14)	1.06(0.95-1.18)	1.07(0.96-1.19)	1.00(0.90-1.12)	0.65
Vitamin B1 (mg)	Ref.	1.03(0.92-1.14)	1.03(0.93-1.15)	0.97(0.87-1.08)	**0.88(0.79-0.98)**	0.01
Vitamin A (mg)	Ref.	1.03(0.93-1.15)	1.05(0.95-1.17)	1.01(0.90-1.12)	**0.90(0.80-1.00)**	0.05
Vitamin B6 (mg)	Ref.	1.02(0.92-1.13)	0.99(0.90-1.11)	1.00(0.90-1.12)	**0.83(0.75-0.93)**	0.0025
Vitamin C (mg)	Ref.	1.00(0.90-1.11)	1.00(0.90-1.11)	0.96(0.87-1.07)	**0.85(0.76-0.95)**	0.005
Vitamin D (mg)	Ref.	0.99(0.90-1.11)	1.05(0.94-1.16)	1.02(0.92-1.14)	0.93(0.84-1.04)	0.34
Vitamin E (mg)	Ref.	1.03(0.93-1.14)	0.95(0.85-1.05)	0.94(0.84-1.04)	**0.83(0.75-0.93)**	0.0003

[a] Dietary inflammatory index® (DII®) score presented by quintile at baseline, which divided the DII® scores into five levels (Q1 to Q5). [b] Data are presented as hazard ratios (HRs) with correspondent 95% confidence intervals (CI). [c] Multivariate-adjusted for gender, age, smoke, alcohol drinking, physical activity, BMI, family history of diabetes mellitus, family history of hypertension and energy intake**.** [d] *p* for trend values were determined using categorical DII® scores. MUFA: monosaturated fatty acid, PUFA, polyunsaturated fatty acid.

Table S3. Hazard ratios (HRs) and 95% confidence intervals (CIs) for risk of metabolic syndrome for selected food parameters according to the DII® quintiles for men participants

	Quintiles of Dietary Inflammatory Index® (DII®)[a,b,c]					
**Food parameters**	Q1	Q2	Q3	Q4	Q5	*P* for trend[d]
**Pro-inflammatory**						
Energy (Kcal)	Ref.	1.23(0.99-1.51)	1.16(0.95-1.43)	**1.36(1.12-1.66)**	**1.22(1.00-1.48)**	0.05
Total fat (g)	Ref.	1.01(0.84-1.21)	1.18(0.99-1.40)	1.04(0.87-1.24)	1.01(0.85-1.21)	0.92
Saturated fat (g)	Ref.	0.94(0.79-1.12)	0.93(0.79-1.11)	0.85(0.71-1.01)	0.98(0.82-1.16)	0.52
Trans fat (g)	Ref.	1.06(0.88-1.27)	1.06(0.88-1.27)	1.05(0.88-1.26)	1.04(0.87-1.24)	0.77
Cholesterol (mg)	Ref.	**0.81(0.68-0.96)**	0.88(0.74-1.03)	0.89(0.75-1.05)	1.01(0.85-1.19)	0.54
Carbohydrate (g)	Ref.	1.08(0.92-1.27)	**1.17(1.00-1.37)**	1.01(0.85-1.20)	1.07(0.89-1.28)	0.59
Protein (g)	Ref.	0.97(0.82-1.16)	1.05(0.89-1.24)	0.96(0.81-1.14)	0.92(0.77-1.09)	0.37
Iron (mg)	Ref.	1.09(0.93-1.28)	1.11(0.95-1.31)	1.05(0.89-1.23)	**0.79(0.66-0.96)**	0.07
Vitamin B12 (mg)	Ref.	1.13(0.96-1.33)	1.15(0.98-1.36)	1.01(0.85-1.20)	1.07(0.90-1.28)	0.83
**Anti-inflammatory**						
Flavan-3-ol (mg)	Ref.	1.04(0.87-1.24)	1.11(0.94-1.30)	0.94(0.80-1.11)	**0.83(0.71-0.98)**	0.0191
Flavones (mg)	Ref.	1.04(0.90-1.21)	1.04(0.89-1.22)	1.08(0.91-1.27)	0.98(0.81-1.19)	0.77
Flavonols (mg)	Ref.	0.98(0.83-1.16)	1.07(0.91-1.26)	0.93(0.80-1.10)	**0.81(0.69-0.96)**	0.0147
Flavonones (mg)	Ref.	1.06(0.91-1.23)	1.15(0.99-1.34)	1.10(0.94-1.30)	0.98(0.80-1.19)	0.56
Tea (g)	Ref.	0.92(0.47-1.78)	1.09(0.94-1.26)	0.93(0.80-1.08)	**0.82(0.70-0.95)**	**0.02**
Iso-flavones (mg)	Ref.	1.22(1.05-1.43)	1.11(0.94-1.30)	1.05(0.89-1.24)	0.85(0.71-1.01)	0.035
MUFA (g)	Ref.	0.98(0.82-1.17)	0.95(0.80-1.13)	0.94(0.79-1.12)	1.02(0.86-1.21)	0.91
PUFA (g)	Ref.	0.97(0.81-1.15)	0.97(0.81-1.15)	0.91(0.76-1.08)	**0.82(0.69-0.98)**	**0.02**
Zinc (mg)	Ref.	1.05(0.89-1.25)	1.07(0.91-1.27)	1.06(0.89-1.25)	1.06(0.90-1.25)	0.55
Caffeine (µg)	Ref.	0.98(0.81-1.19)	1.08(0.90-1.30)	1.07(0.89-1.30)	1.11(0.92-1.34)	0.11
Carotene (µg)	Ref.	0.94(0.80-1.10)	1.00(0.84-1.17)	1.04(0.88-1.22)	0.91(0.76-1.08)	0.73
Fiber (g)	Ref.	0.89(0.76-1.03)	0.90(0.76-1.05)	0.97(0.82-1.14)	**0.80(0.67-0.96)**	0.08
Folic acid (mg)	Ref.	0.89(0.76-1.04)	0.87(0.74-1.02)	0.94(0.80-1.11)	0.84(0.70-1.00)	0.12
Garlic (g)	Ref.	0.93(0.78-1.12)	0.98(0.82-1.17)	1.00(0.84-1.19)	0.96(0.80-1.14)	0.95
Magnesium (mg)	Ref.	1.03(0.88-1.21)	1.10(0.93-1.29)	1.08(0.91-1.27)	0.85(0.71-1.02)	0.33
N-3 PUFA (g)	Ref.	0.99(0.84-1.17)	0.95(0.80-1.13)	1.02(0.87-1.21)	0.84(0.70-1.00)	0.13
N-6 PUFA (g)	Ref.	1.19(0.98-1.44)	1.22(1.01-1.47)	1.16(0.96-1.40)	1.14(0.94-1.37)	0.43
Niacin (mg)	Ref.	1.24(1.04-1.48)	1.13(0.95-1.35)	1.17(0.98-1.39)	1.04(0.87-1.24)	0.97

Onion (g)	Ref.	0.95(0.80-1.13)	1.16(0.98-1.37)	1.21(1.03-1.43)	1.16(0.97-1.38)	**0.004**
Riboflavin (mg)	Ref.	0.95(0.81-1.12)	0.98(0.83-1.15)	0.98(0.83-1.15)	**0.81(0.67-0.97)**	0.09
Anthocyanidins (mg)	Ref.	1.06(0.91-1.23)	1.07(0.92-1.25)	1.04(0.88-1.22)	0.99(0.82-1.19)	0.98
Selenium (mg)	Ref.	1.04(0.86-1.25)	1.01(0.84-1.21)	1.06(0.88-1.27)	0.98(0.82-1.17)	0.84
Vitamin B1 (mg)	Ref.	1.09(0.91-1.29)	1.07(0.90-1.27)	1.05(0.88-1.24)	1.05(0.88-1.24)	0.77
Vitamin A (mg)	Ref.	0.92(0.78-1.08)	1.05(0.89-1.23)	1.01(0.86-1.19)	0.88(0.74-1.05)	0.54
Vitamin B6 (mg)	Ref.	1.06(0.91-1.24)	1.00(0.86-1.18)	0.99(0.84-1.16)	0.91(0.77-1.09)	0.25
Vitamin C (mg)	Ref.	0.89(0.77-1.04)	0.95(0.82-1.11)	1.00(0.85-1.18)	0.83(0.69-1.01)	0.32
Vitamin D (mg)	Ref.	1.00(0.86-1.18)	0.99(0.84-1.17)	1.01(0.86-1.19)	0.89(0.75-1.07)	0.31
Vitamin E (mg)	Ref.	1.21(1.04-1.41)	0.97(0.82-1.14)	0.89(0.75-1.06)	**0.91(0.76-1.09)**	**0.01**

[a] Dietary inflammatory index® (DII®) score presented by quintile at baseline, which divided the DII® scores into five levels (Q1 to Q5). [b] Data are presented as hazard ratios (HRs) with correspondent 95% confidence intervals (CI). [c] Multivariate-adjusted for age, smoke, alcohol drinking, physical activity, BMI, family history of diabetes mellitus, family history of hypertension and energy intake. [d] *p* for trend values were determined using categorical DII® scores. MUFA: monosaturated fatty acid, PUFA, polyunsaturated fatty acid.

Table S4. Hazard ratios (HRs) and 95% confidence intervals (CIs) for risk of metabolic syndrome for selected food parameters according to the DII® quintiles for women participants

Food parameters	Quintiles of Dietary Inflammatory Index® (DII®)[a,b,c]					*P* for trend[d]
	Q1	Q2	Q3	Q4	Q5	
**Pro-inflammatory**						
Energy (Kcal)	Ref.	0.93(0.81-1.05)	0.99(0.87-1.13)	0.96(0.84-1.10)	**0.83(0.72-0.96)**	0.05
Total fat (g)	Ref.	0.97(0.85-1.10)	1.07(0.94-1.22)	0.88(0.76-1.01)	0.98(0.85-1.13)	0.43
Saturated fat (g)	Ref.	1.02(0.89-1.17)	1.09(0.95-1.24)	0.98(0.85-1.12)	0.94(0.82-1.08)	0.37
Trans fat (g)	Ref.	0.99(0.87-1.12)	0.93(0.82-1.06)	0.97(0.85-1.12)	1.04(0.90-1.19)	0.84
Cholesterol (mg)	Ref.	0.95(0.83-1.08)	0.96(0.84-1.10)	1.05(0.92-1.21)	0.90(0.78-1.03)	0.54
Carbohydrate (g)	Ref.	1.00(0.86-1.16)	1.07(0.93-1.24)	1.04(0.90-1.20)	1.07(0.93-1.24)	0.26
Protein (g)	Ref.	0.91(0.80-1.05)	0.94(0.82-1.08)	0.94(0.82-1.07)	**0.85(0.74-0.97)**	0.05
Iron (mg)	Ref.	1.04(0.90-1.19)	1.01(0.88-1.16)	0.95(0.83-1.10)	**0.79(0.69-0.91)**	0.0004
Vitamin B12 (mg)	Ref.	1.05(0.91-1.20)	1.01(0.88-1.16)	1.01(0.88-1.16)	0.95(0.83-1.09)	0.41
**Anti-inflammatory**						
Flavan-3-ol (mg)	Ref.	1.12(0.97-1.28)	1.06(0.92-1.22)	0.89(0.77-1.02)	**0.82(0.71-0.95)**	<.0001
Flavones (mg)	Ref.	1.16(0.99-1.34)	1.20(1.04-1.39)	1.16(1.00-1.33)	1.02(0.88-1.17)	0.89
Flavonols (mg)	Ref.	1.03(0.90-1.18)	1.03(0.90-1.19)	**0.85(0.74-0.98)**	**0.78(0.67-0.90)**	<.0001
Flavonones (mg)	Ref.	1.12(0.97-1.30)	1.21(1.05-1.39)	1.13(0.98-1.30)	1.09(0.94-1.25)	0.40
Tea (g)	Ref.	0.69(0.35-1.33)	0.98(0.87-1.10)	**0.83(0.73-0.93)**	**0.76(0.67-0.86)**	<0.0001
Iso-flavones (mg)	Ref.	1.16(1.01-1.34)	1.06(0.92-1.23)	0.95(0.82-1.10)	0.88(0.76-1.02)	0.0017
MUFA (g)	Ref.	1.01(0.88-1.15)	1.01(0.88-1.15)	1.09(0.95-1.25)	0.96(0.83-1.10)	0.99
PUFA (g)	Ref.	0.94(0.82-1.07)	0.98(0.85-1.12)	0.94(0.82-1.08)	**0.81(0.71-0.94)**	0.01
Zinc (mg)	Ref.	1.03(0.90-1.18)	1.00(0.88-1.15)	0.94(0.82-1.10)	0.94(0.82-1.10)	0.19
Caffeine (µg)	Ref.	1.01(0.88-1.16)	1.18(1.03-1.35)	1.11(0.97-1.28)	1.29(1.12-1.49)	0.0001
Carotene (µg)	Ref.	1.12(0.97-1.29)	1.12(0.97-1.28)	0.98(0.85-1.12)	0.94(0.82-1.08)	0.08
Fiber (g)	Ref.	1.00(0.86-1.15)	1.03(0.89-1.18)	0.92(0.80-1.06)	**0.82(0.71-0.95)**	0.001
Folic acid (mg)	Ref.	1.06(0.92-1.22)	1.01(0.88-1.17)	0.92(0.80-1.06)	0.87(0.76-1.01)	0.007
Garlic (g)	Ref.	0.95(0.83-1.10)	1.09(0.96-1.24)	0.96(0.84-1.09)	0.98(0.86-1.11)	0.79
Magnesium (mg)	Ref.	1.10(0.95-1.25)	1.06(0.93-1.22)	1.15(1.00-1.32)	1.03(0.90-1.18)	0.48
N-3 PUFA (g)	Ref.	1.00(0.87-1.14)	0.99(0.86-1.13)	1.04(0.91-1.19)	0.88(0.77-1.00)	0.16
N-6 PUFA (g)	Ref.	1.07(0.94-1.21)	1.11(0.97-1.26)	0.98(0.85-1.13)	1.06(0.92-1.23)	0.67
Niacin (mg)	Ref.	1.10(0.96-1.26)	1.10(0.96-1.26)	1.06(0.92-1.22)	0.98(0.85-1.13)	0.63

Onion (g)	Ref.	1.08(0.93-1.24)	1.11(0.97-1.29)	1.26(1.10-1.45)	1.13(0.99-1.30)	0.01
Riboflavin (mg)	Ref.	0.96(0.84-1.10)	0.95(0.83-1.09)	0.91(0.79-1.04)	**0.82(0.71-0.94)**	0.003
Anthocyanidins (mg)	Ref.	1.15(0.99-1.34)	1.12(0.97-1.30)	1.20(1.04-1.38)	1.16(1.01-1.34)	0.0443
Selenium (mg)	Ref.	1.00(0.88-1.15)	1.08(0.94-1.23)	1.06(0.92-1.21)	1.00(0.87-1.15)	0.69
Vitamin B1 (mg)	Ref.	0.99(0.87-1.13)	1.02(0.89-1.16)	0.92(0.80-1.06)	**0.78(0.68-0.90)**	0.001
Vitamin A (mg)	Ref.	1.12(0.98-1.29)	1.05(0.92-1.21)	1.01(0.88-1.16)	0.91(0.79-1.05)	0.05
Vitamin B6 (mg)	Ref.	0.99(0.86-1.14)	0.99(0.86-1.14)	1.00(0.88-1.15)	**0.80(0.69-0.92)**	0.0034
Vitamin C (mg)	Ref.	1.11(0.96-1.28)	1.04(0.90-1.20)	0.97(0.84-1.12)	0.88(0.76-1.01)	0.006
Vitamin D (mg)	Ref.	0.98(0.85-1.13)	1.09(0.95-1.25)	1.03(0.90-1.19)	0.95(0.83-1.09)	0.68
Vitamin E (mg)	Ref.	0.90(0.79-1.04)	0.93(0.81-1.07)	0.96(0.84-1.10)	**0.79(0.69-0.91)**	0.009

[a] Dietary inflammatory index® (DII®) score presented by quintile at baseline, which divided the DII® scores into five levels (Q1 to Q5). [b] Data are presented as hazard ratios (HRs) with correspondent 95% confidence intervals (CI). [c] Multivariate-adjusted for age, smoke, alcohol drinking, physical activity, BMI, family history of diabetes mellitus, family history of hypertension and energy intake. [d] *p* for trend values were determined using categorical DII® scores. MUFA: monosaturated fatty acid, PUFA, polyunsaturated fatty acid.
